# Survival and determinants of mortality in adult HIV/Aids patients initiating antiretroviral therapy in Somali Region, Eastern Ethiopia

**DOI:** 10.11604/pamj.2015.22.138.4352

**Published:** 2015-10-14

**Authors:** Bereket Damtew, Bezatu Mengistie, Tadesse Alemayehu

**Affiliations:** 1 Department of Epidemiology and Biostatistics, College of Health and Medical Sciences, Haramaya University, East Harerge, Ethiopia; 2 Department of Public Health, College of Health and Medical Sciences, Haramaya University, East Harerge, Ethiopia; 3 Department of Public Health, College of Health and Medical Sciences, Haramaya University, East Harerge, Ethiopia

**Keywords:** HIV, Aids, antiretroviral therapy, Ethiopia

## Abstract

**Introduction:**

Studies have shown high initial mortality in Antiretroviral Therapy (ART) programs from resource-limited settings. However, there is dearth of evidence on treatment outcomes and associated determinant factors in public hospitals. Therefore, the objective of this study is to assess survival and identify predictors of death in adult HIV-infected patients initiating ART at a public hospital in Eastern Ethiopia.

**Methods:**

A retrospective cohort study was conducted by reviewing baseline and follow-up records of patients who started ART between December 1, 2007 and December 31, 2011 at Kharamara hospital. Time to death was the main outcome measure. Kaplan-Meier models were used to estimate mortality and Cox proportional hazards models to identify predictors of mortality.

**Results:**

A total of 784 patients (58.4% females) were followed for a median of 60 months. There were 87 (11.1%) deaths yielding an overall mortality rate of 5.15/100 PYO (95% CI: 4.73-6.37). The estimated mortality was 8.4%, 9.8%, 11.3%, 12.7% and 14.1% at 6, 12, 24, 36 and 48 months respectively. The independent predictors of death were single marital status (AHR: 2.31; 95%CI: 1.18-4.50), a bedridden functional status (AHR: 5.91; 95%CI: 2.87-12.16), advanced WHO stage (AHR: 7.36; 95%CI: 3.17-17.12), BMI < 18.5 Kg/m2 (AHR: 2.20; 95%CI: 1.18-4.09), CD4 count < 50 cells/µL (AHR: 2.70; 95%CI: 1.26-5.80), severe anemia (AHR: 4.57; 95%CI: 2.30-9.10), and TB co-infection (AHR: 2.30; 95%CI: 1.28-4.11).

**Conclusion:**

Improved survival was observed in patients taking ART in Somali region of Ethiopia. The risk for death was higher in patients with advanced WHO stage, low CD4 count, low Hgb, low BMI, and concomitant TB infection. Intensive case management is recommended for patients with the prognostic factors. Optimal immunologic and weight recoveries in the first 6 months suggest increased effort to retain patients in care at this period.

## Introduction

According to the 2012 UNAIDS report, there are an estimated 34 million People Living with HIV (PLHIV) worldwide, Sub-Saharan Africa (SSA) accounting for 69%, with nearly 1 in every 20 adults (4.9%) living with HIV; Around 1.7 million people died from AIDS-related causes worldwide, 70% occurred in SSA [1]. Ethiopia is one of the hardest hit sub-Saharan African countries by the HIV pandemic. By the end of 2012, there were 759,268 people living with HIV; 41,444 deaths from AIDS-related causes and 20,158 new HIV infections [2]. For 2007, the estimated life expectancy was 52.5 years, with 3.9 years lost to AIDS [3]. In January 2005, the FDRE Ministry of Health (MoH) launched the free ART rollout program. By the end of 2011 there were 249,174 patients on treatment, making the ART coverage for adult population of PLHIV 86% [4]. The introduction of HAART witnessed a decrease in AIDS-defining opportunistic infections, a decline in AIDS related mortalities, and improved survival of PLHIV. Despite increased availability of ARV and promising efficacy reported from ART programs in resource-limited settings, mortality has been high particularly the first few months after initiating ART [5, 6]. Reporting treatment outcomes of patients enrolled in ART programs is important to demonstrate program effectiveness and justify continued funding, while assessment of factors associated with outcomes can help to identify opportunities for program improvement [7]. Few studies have reported baseline socio-demographic and clinical factors in predicting survival after ART initiation [8–10]. However, there are no studies describing mortality on ART and its associated factors over longer follow up periods. Therefore, this study determines factors associated with survival and progressive immunologic and weight changes among adult PLHIV receiving ART in Somali region, Ethiopia.

## Methods

**Study setting:** the study was conducted at Kharamara hospital, in Jijiga town from March to May 2013. Jijiga, the administrative capital of Somali region, is located 614 km to the east of Addis Ababa, Ethiopia's capital. The hospital provides general outpatient and inpatient services, surgical and obstetric emergency, and regional referral hospital for ophthalmologic service. In the comprehensive HIV care and treatment service at the hospital, there are 9,208 patients enrolled, of which 1,501 ever-started ART, and 1,432 currently on ART.

**Study design:** a retrospective cohort study was conducted to assess survival and determinants of mortality among PLHIV receiving antiretroviral therapy. A total of 784 PLHIV aged ≥15 years, initiating ART at Kharamara hospital between December 1, 2007 and December 31, 2011 were included in the study. The independent variables were socio-demographic characteristics, baseline clinical, laboratory and ART information. The main outcome measure in the study was follow-up time till Death. The second outcome measures were median changes in CD4 count and weight.

**Participants:** the source population was adult HIV/AIDS patients who started ARV and were on follow up at the ART clinic during the study period. Patients who initiated treatment outside Kharamara hospital, women who were pregnant and lactating mothers on PMTCT, and patients with competing causes of death (accident, or any non-AIDS malignancies) were excluded. The sample size was determined by taking the mortality rates in two groups of PLHIV on ART based on their WHO clinical stage as exposure status. The mortality rate among exposed (WHO stage IV) is 0.3% and among non-exposed (WHO stage II-III), 0.1% [10]. The study participants were randomly selected using patients’ unique identification number and were retrospectively followed for additional 60 months until December 31, 2012.

**Data collection and quality control:** to compile the required information a data collection form was developed from the national ART intake and follow-up forms. The data were collected by reviewing pre-ART register, ART intake form, laboratory request, monthly cohort form, and follow up form. The most recent laboratory results before starting ART were used as a baseline value. If there were no pre-treatment laboratory tests, results obtained within one month of ART initiation were used as baseline. If two results were obtained within a month, their mean would be used. Death from all AIDS related causes was ascertained by reviewing patients’ medical records in the hospital, or registrations reported by adherence supporters. The data were collected by two experienced ART nurses who were trained on comprehensive HIV care and who are working in the ART clinic at Kharamara hospital. A supervisor supervised the data collection process. The data collectors and the supervisor were trained on the data collection procedures for two days. The investigator oversaw the overall process. All completed data collection forms were examined for completeness, consistency and clarity during data management, storage, and analysis.

**Data analysis:** data exploration was carried out to check for any inconsistencies, coding error, out of range, and missing values and appropriate corrections were made. Descriptive analyses of the continuous and categorical data describing the cohort's characteristics at baseline and during follow-up were made. Patients were censored on the date of any one of the following events, whichever occurred first: if lost to follow-up, if transferred to another health facility, or if alive at the end of follow up. The outcomes of each patient were dichotomized into censored or dead. Kaplan-Meier model was be used to assess survival functions stratified by baseline and follow up variables, and the log-rank test was used to assess statistical difference among groups (for equality of survival distributions). Cox-proportional Hazards model was used to identify prognostic factors of death, and to calculate their bivariate and adjusted hazard ratios. All analyses were conducted using SPSS version 16.0 for windows (SPSS^®^ Inc., Chicago, IL, USA).

## Results

The study cohort included 784 patients with a mean age of 34 (SD±10). Out of the total study population, 485 (58.4%) were females. Three hundred twenty five (41.5%) were married and 455 (58%) had dependent children at home. Four hundred eighty seven (62.1%) had at least completed primary education, and 359 (45.8%) had no occupation. The majority 431 (55%) had good ART adherence (see Table 1). At the time of ART initiation, 455 (58.0%) were in WHO clinical stage III&IV and 242 (30.9%) had an ambulatory functional status. Among the study participants, 449 (57.3%) had a BMI < 18.5 Kg/m^2^ and 311 (39.7%) developed active TB infection. With regard to chemoprophylaxis, the majority 658 (83.9%) were given cotrimoxazole and only 61 (7.8%) received INH. The median (IQR) weight, CD4 count and hemoglobin of the cohort were 50kg (44-56), 146 cells /µL (102-196) and 11.5 g/dL (10-13) respectively (see Table 2).

**Table 1:** Socio-demographic characteristics of adult PLHIV receiving ART in Somali region, Ethiopia (N = 784), May 2013

Variables	Total (N,%)	Dead (N,%)	Active (N,%)	Log-rank *P*
***Sex***				
Male	326 (41.6%)	46 (52.9%)	280 (40.2%)	0.019
Female	458 (58.4%)	41 (47.1%)	417 (59.8%)	
***Age category***				
15-24	108 (13.8%)	6 (6.9%)	102 (14.6%)	0.249
25-34	327 (41.7%)	41 (47.1%)	102 (14.6%)	
35-44	229 (29.2%)	25 (28.7%)	204 (29.3%)	
45+	120 (15.3%)	15 (17.2%)	105 (15.1%)	
***Religion***				
Muslim	319 (40.7%)	35 (11.0%)	284 (89.0%)	0.714
Orthodox	438 (55.9%)	50 (11.4%)	388 (88.6%)	
Protestant	27 (3.4%)	2 (7.4%)	25 (92.6%)	
***Marital status***				
Married	325 (41.5%)	27 (31.0%)	298 (42.8%)	0.002
Single	115 (14.7%)	24 (27.6%)	91 (13.1%)	
Separated	27 (3.4%)	1 (1.1%)	26 (3.7%)	
Divorced	226 (28.8%)	26 (29.9%)	200 (28.7%)	
Widowed	91 (11.6%)	9 (10.3%)	82 (11.8%)	
***Educational status***				
No education	297 (37.9%)	48 (55.2%)	249 (35.7%)	0.000
Primary	217 (27.7%)	27 (31.0%)	190 (27.3%)	
Secondary	207 (26.4%)	10 (11.5%)	197 (28.3%)	
College/Above	63 (8.0%)	2 (2.3%)	61 (8.8%)	
***Dependent children***				
Yes	455 (58.0%)	52 (59.8%)	403 (57.8%)	0.889
No	329 (42.0%)	35 (40.2%)	294 (42.2%)	
***ART Adherence***				
Good	431 (55.0%)	36 (41.4%)	395 (56.7%)	0.000
Fair	431 (55.0%)	20 (23.0%)	157 (22.5%)	
Poor	176 (22.4%)	31 (35.6%)	145 (20.8%)	

**Table 2:** Clinical and laboratory markers of adult PLHIV receiving ART in Somali region, Ethiopia (N = 784), May 2013

Variables	Total (N,%)	Dead (N,%)	Active (N,%)	Log-rank *P*
***Functional status***				
Working	434 (55.4%)	12 (13.8%)	422 (60.5%)	0.000
Ambulatory	242 (30.9%)	37 (42.5%)	205 (29.4%)	
Bedridden	108 (13.8%)	38 (43.7%)	70 (10.0%)	
***WHO staging***				
Stage I & II	329 (42.0%)	10 (11.5%)	319 (45.8%)	0.000
Stage III	327 (41.7%)	38 (43.7%)	289 (41.5%)	
Stage IV	128 (16.3%)	39 (44.8%)	89 (12.8%)	
***BMI for age***				
≥18.5 Kg/m^2^	335 (42.7%)	23 (26.4%)	312 (44.8%)	0.000
< 18.5 Kg/m^2^	449 (57.3%)	64 (73.6%)	385 (55.2%)	
***Cotrimoxazole***				
Given	658 (83.9%)	66 (75.9%)	592 (84.9%)	0.032
Not given	126 (16.1%)	21 (24.1%)	105 (15.1%)	
***INH Given***				
Given	61 (7.8%)	20 (23.0%)	41 (5.9%)	0.000
Not given	723 (92.2%)	67 (77.0%)	656 (98.3%)	
***TB co-infected***				
Yes	311 (39.7%)	45 (51.7%)	266 (38.2%)	0.015
No	473 (60.3%)	42 (48.3%)	431 (61.8%)	
***Weight (Kg)*** ^***+***^	50 (44-56)	43 (37-50)	51 (45-56)	0.000
***CD4 (cells/ µL)*** ^***+***^	146 (102-196)	48 (31-99)	156 (116-206)	0.000
***Hemoglobin (g/dL)*** ^***+***^	11.5 (10.0-13.0)	9.4 (7.9-11.3)	11.8 (10.3-13.1)	0.000

+ Median value (25th-75th percentile)

### Follow up

**Figure 1:**
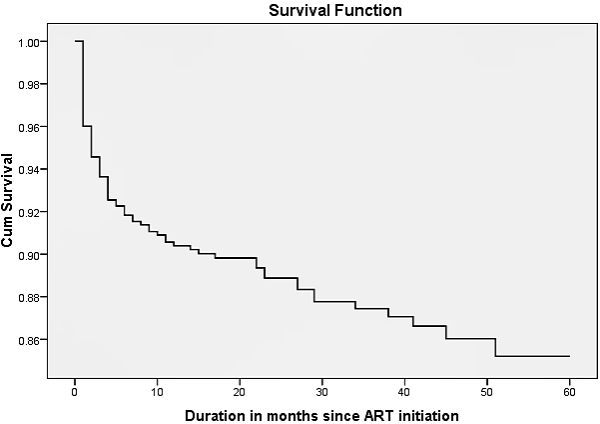
Cumulative survival of adult PLHIV receiving ART in Somali region, Ethiopia, May 2013

A total of 87 (11.1%) patients died during the five year follow-up period, with majority of deaths 49(56.3%) occurring in the first 3 months (HR: 0.022). One hundred fifty seven (20.0%) patients were transferred to other facility and 193 (24.6%) were lost-to-follow up. The remaining 344 (43.9%) were active until the last censoring date. The median survival time for event (death) was 20.7 months (IQR, 17.5-22.6). The overall mortality rate in the cohort during the 1,608 person-years of observation (PYO) was 5.15/100 PYO (95% CI: 4.73-6.37). The estimated mortality was 8.4%, 9.8%, 11.3%, 12.7% and 14.1% at 6, 12, 24, 36 and 48 months respectively (see Figure 1).

### Predictors of mortality

In bivariate Cox regression analysis, sex, marital status, education level, functional status, WHO clinical stage, BMI, CD4 count, Anemia, cotrimoxazole prophylaxis, INH prophylaxis, TB co-infection, and ART adherence were all associated with survival (P < 0.05). In the multivariate Cox regression analysis, the independent, significant predictors of mortality in PLHIV on ART at Kharamara hospital were single marital status (AHR: 2.31; 95%CI: 1.18-4.50), a bedridden functional status (AHR: 5.91; 95%CI: 2.87-12.16), advanced WHO stage (AHR: 7.36; 95%CI: 3.17-17.12), BMI < 18.5 Kg/m2 (AHR: 2.20; 95%CI: 1.18-4.09), CD4 count < 50 cells/µL (AHR: 2.70; 95%CI: 1.26-5.80), severe anemia (AHR: 4.57; 95%CI: 2.30-9.10), and TB co-infection (AHR: 2.30; 95%CI: 1.28-4.11) (see Table 3).

**Table 3:** Predictors of mortality among adult PLHIV receiving ART in Somali region, Ethiopia (N = 784), May 2013

Variables	Crude HR (95% CI)	*P* value	AHR (95% CI)	*P* value
***Marital status***				
Married	1	-	1	-
Single	2.69 (1.55, 4.66)	0.001	2.31 (1.18, 4.50)	0.015
Separated	0.40 (0.05, 2.94)	0.367	0.13 (0.014, 1.21)	0.073
Divorced	1.41 (0.82, 2.41)	0.215	1.47 (0.80, 2.71)	0.215
Widowed	1.15 (0.54, 2.44)	0.719	1.27 (0.56, 2.86)	0.566
***Educational status***				
No education	5.66 (1.38, 23.29)	0.016	4.73 (1.07, 22.40)	0.038
Primary	4.26 (1.01, 17.91)	0.048	3.83 (0.80, 18.30)	0.082
Secondary	1.54 (0.34, 7.05)	0.575	2.43 (0.50, 12.01)	0.281
College/Above	1	-	1	-
***Functional status***				
Working	1	-	1	-
Ambulatory	6.16 (3.21, 11.82)	0.000	2.12 (1.05, 4.26)	0.035
Bedridden	15.42 (8.05, 29.53)	0.000	5.91 (2.87, 12.16)	0.000
***WHO staging***				
Stage I&II	1	-	1	-
Stage III	5.28 (2.60, 10.71)	0.000	4.00 (1.83, 8.74)	0.000
Stage IV	16.22 (7.93, 33.20)	0.000	7.36 (3.17, 17.12)	0.000
***BMI for age***				
≥18.5 Kg/m^2^	1	-	1	
< 18.5 Kg/m^2^	2.27 (1.41, 3.66)	0.001	2.20 (1.18, 4.09)	0.013
***CD4 category***				
≥ 200	1	-	1	-
125-200	3.87 (2.05, 7.27)	0.000	1.33 (0.65, 2.72)	0.440
50-125	5.62 (3.04, 10.41)	0.000	2.24 (1.08, 4.65)	0.031
< 50	17.36 (9.96, 30.28)	0.000	2.70 (1.26, 5.80)	0.011
***Anemia***				
Normal	1	-	1	-
Mild	1.16 (0.56, 2.42)	0.695	1.16 (0.52, 2.60)	0.724
Moderate	3.27 (1.69, 6.32)	0.000	2.90 (1.36, 6.16)	0.006
Severe	8.01 (4.53, 14.17)	0.000	4.57 (2.30, 9.10)	0.000
***TB co-infected***				
Yes	2.21 (1.35, 3.62)	0.002	2.30 (1.28, 4.11)	0.005
No	1	-	1	-

### Weight and CD4 change during the follow-up

**Figure 2:**
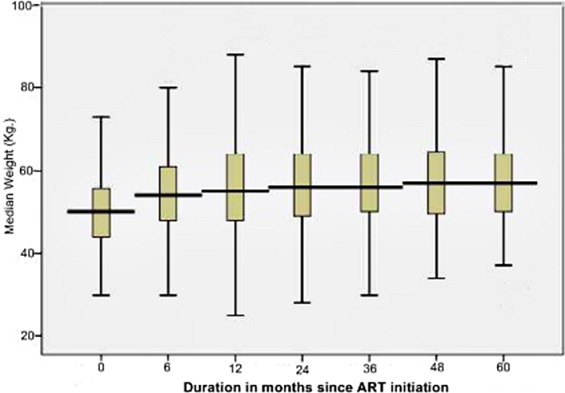
Median weight change during follow-up for adult PLHIV receiving ART in Somali region, Ethiopia, May 2013

**Figure 3:**
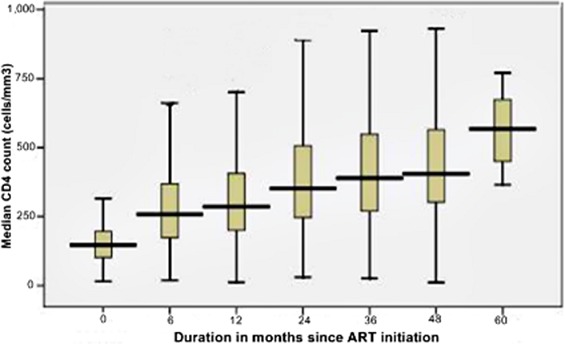
Median CD4 change during follow-up for adult PLHIV receiving ART in Somali region, Ethiopia, May 2013

Weight and CD4 recovery were used as supplementary indicators for comprehensive treatment outcomes. At the start of ART, the median weight of the cohort was 50.0 Kg. During the follow-up period, the median weight (IQR) at 6, 12, 24, 36, 48 and 60 months were 54 (48-61), 55 (48-64), 56 (50-64), 56 (49-64), 57 (50-65) and 57 (50-64), Kg., respectively (Figure 2). Similarly, the median CD4 count at baseline was 146cells/µL. In the follow-up period, the median CD4 count (IQR) changed to 258 (172-369), 286 (201-406), 351 (246-506), 389 (270-549), 405 (302-565) and 567 (390-770) at 6, 12, 24, 36, 48 and 60 months, respectively (Figure 3). During the follow-up period, 123 patients had their CD4 count declined to below or equal to the baseline values yielding an immunologic treatment failure rate of 7.84/100 PYO (95%CI:6.89-8.81).

## Discussion

In this historical longitudinal study, there were 87 deaths in 1,608 PYO, yielding an incidence density of 5.15/100 PYO (95%CI: 4.73-6.37). The independent predictors of mortality include single marital status, being illiterate, bedridden functional status, advanced WHO status, low BMI, low CD4 count, severe anemia and TB co-infection. The estimated survival probability of the cohort at 6, 12, 24, 36 and 48 months was 91.6%, 90.2%, 88.7%, 87.3% and 85.9%, respectively. This shows a better survival compared to other studies in Africa. According to a study in a Malawian cohort, the probability of being alive on ART at 6, 12 and 18 months was 89.8%, 83.4% and 78.8% respectively [11]. On the other hand, the death rate was comparable to most studies, especially, in the 1st six months [6, 8, 9]. This might be explained by the fact that most of the patients in this study had advanced disease status (78% had CD4 < 200 cells/mL and 58% were in WHO stage III & IV). Patients who were illiterate had high risk of mortality compared to those with college education or above (AHR: 4.73; 95%CI: 1.07-22.40). A study conducted in Ethiopia also found a strong association between level of education and survival [12]. Single patients had a higher risk for death compared to married patients (AHR: 2.31; 95%CI: 1.18-4.50). This difference might be due to married patients’ psychological preparedness to seek partners’ and social support, conceive the facts, and adhere to ART [13].

The result from this study shows patients with a CD4 count of < 50 cells/mL have a higher risk of mortality (AHR: 2.70; 95%CI: 1.26-5.80) compared to those with a CD4 count of ≥ 200 cells/mL. Majority of previous studies also found twice or more risk of mortality in patients with lower CD4 count compared to those with a CD4 count of ≥ 200 cells/mL [5, 12, 14]. Patients with severe anemia were 4.57 more at risk of death compared to those with normal levels. In a study from Tanzania, patients with severe anemia were 15 times higher at risk of dying during the first year on ART compared to those with a normal hemoglobin level [6]. Other studies have also indicated patients with low hemoglobin level (<10 mg/dL) had increased risk of death [5, 9, 15]. Although there is no concrete evidence on casual association between anemia and mortality, the incidence of anemia increased with progression of HIV disease [14]. In this study also, severe anemia was associated with an advanced WHO stage (24.2% in Stage IV vs. 8.8% in stage I&II). One of the most important side effects of AZT is myelotoxicity leading to severe anemia [15]. In this cohort, severe anemia in patients taking AZT was higher (46.7%) among the dead compared to those censored (8.8%). In the current study, the risk of death in patients with a BMI < 18.5 Kg/m^2^ was more than two times higher (AHR: 2.20; 95%CI: 1.18-4.09) compared to those with a BMI < 18.5kg/m^2^. Study conducted in rural Malawi showed individuals who were severely malnourished (BMI < 16kg/m^2^) had six times higher risk of dying in the first three months than those with a normal nutritional status [16]. BMI is an indicator of patient nutritional status but may also be influenced by late-stage AIDS conditions, such as wasting syndrome and opportunistic infections, or by progression of the HIV itself [17]. In the current cohort, there was a significant difference in mean BMI between TB +VE patients (18.10; 95%CI: 17.66-18.54) and TB -VE ones (18.77; 95%CI: 18.40-19.14), and also, between WHO stage I&II (19.68; 95%CI: 19.24-20.13) and WHO stage IV (17.13; 95%CI: 16.49-17.77). TB co-infection at baseline or later was also associated with increased risk of mortality (AHR: 2.30; 95%CI: 1.28-4.11). Study conducted in Uganda shows, after adjusting for a history of HIV related infections, the overall relative hazard for death associated with tuberculosis was 1.81 (95%CI: 1.24-2.65) [18]. Manosuthi et.al also showed patients who delayed ART for > 6 months after TB diagnosis had a higher mortality rate than those who initiated ART < 6 months after TB diagnosis [19]. Other studies in Ethiopia have also showed the similar relationship [10, 20]. High mortality in patients living with HIV/AIDS in poor countries was linked to concomitant TB infection [15]. This may be because TB is the leading cause of death worldwide, and the virulence of the mycobacterium increases in HIV infected patients, where the host's immune system is suppressed, enabling it to establish infection very easily [21].

Different studies conducted in African countries, including Ethiopia, have shown a progressive change in CD4 count and weight after initiation of ART [9, 22]. These studies showed that the most significant increment in the median CD4 count occurred in the first six months of ART which is also the case in the current study; wherein a 76.2% increase from baseline median level was observed in the 1st six months. The recovery in weight also showed a similar progress in the 1st six months (8% gains). However, the change in median CD4 and median weight in subsequent months was minimal, with an average gain of 17% and 1.4%, respectively. In addition, the immunologic recovery was much slower in patients with baseline AIDS defining disease [18].

## Conclusion

Improved survival was observed in patients taking ART in Somali region of Ethiopia. However, the risk for death was higher in patients with bed-ridden functional status, advanced WHO stage, low CD4 count, severe anemia, low BMI, and concomitant TB infection. Intensive clinical and nutritional rehabilitation is recommended during the earliest follow up periods on ART for patients with these prognostic factors. Providing screening and chemoprophylaxis for TB to patients on ART is insurmountable in deterring the infection and decreasing mortality. Optimal immunologic and weight recoveries occurred in the first six months indicating the need for patient retention, esp. during this crucial period.
